# Teclistamab in relapsed/refractory light chain amyloidosis: A retrospective multicenter study by the German Society for Amyloid Diseases

**DOI:** 10.1002/hem3.70389

**Published:** 2026-06-16

**Authors:** Alexander Carpinteiro, Christoph Kimmich, Despina Trajanova, Ute Hegenbart, Timon Hansen, Udo Holtick, Vera von Landenberg‐Roberg, Stephan Rainer Bohl, Raphael Teipel, Ivana von Metzler, Monika Engelhardt, Evgenii Shumilov, Maximilian Steinhardt, Hans Christian Reinhardt, Sara Oubari, Stefan Schönland

**Affiliations:** ^1^ Department of Hematology and Stem Cell Transplantation West German Cancer Center, University Hospital Essen Essen Germany; ^2^ Institute of Molecular Biology University of Duisburg‐Essen Essen Germany; ^3^ West German Amyloidosis Center University Hospital Essen Essen Germany; ^4^ Department of Oncology and Hematology Klinikum Oldenburg, University Clinic Oldenburg Germany; ^5^ Department of Internal Medicine V, Amyloidosis Center Heidelberg University Hospital Heidelberg Heidelberg Germany; ^6^ Onkologicum HOPA Hamburg Germany; ^7^ Department I of Internal Medicine, Medical Faculty and University Hospital of Cologne University of Cologne Cologne Germany; ^8^ Department of Hematology and Cancer Immunology, Amyloidosis Center Charité Berlin, Charité ‐ University Medicine Berlin Freie Universität and Humboldt‐Universität zu Berlin Berlin Germany; ^9^ Department of Internal Medicine I University Hospital Carl Gustav Carus TU Dresden Dresden Germany; ^10^ Department of Medicine II – Hematology and Oncology, University Hospital Frankfurt Goethe‐University Frankfurt Frankfurt am Main Germany; ^11^ Department of Hematology and Oncology, University Hospital Freiburg Faculty of Freiburg Freiburg Germany; ^12^ Department of Hematology and Stem Cell Transplantation University Hospital Münster Münster Germany; ^13^ Medizinsiche Klinik und Poliklinik 2 University Hospital Würzburg Würzburg Germany; ^14^ Interdisciplinary Amyloidosis Center Würzburg Würzburg Germany; ^15^ German Cancer Consortium (DKTK) Partner Site University Hospital Essen Essen Germany

## Abstract

Systemic light chain (AL) amyloidosis is a rare, acquired protein misfolding disorder characterized by extracellular deposition of misfolded immunoglobulin light chain fibrils, resulting in organ damage. Treatment is based on anti‐plasma cell regimens derived from multiple myeloma therapy. To date, no approved regimens exist for relapsed/refractory cases. This retrospective multicenter study, performed at 11 centers across Germany, evaluated the efficacy and tolerability of the treatment with teclistamab in 52 patients with relapsed/refractory AL amyloidosis. Hematologic response (≥very good partial response [VGPR]) was achieved in 81% of patients at Day 15, which increased to 95% at 3 months. Flow‐minimal residual disease (MRD) was negative in 96% (23/24) of cases. Cardiac and renal responses (≥partial response [PR]) at 6 months were 65% and 78%, respectively. Cytokine release syndrome occurred in 37% of cases, with two classified as Grade 3 and none as Grade 4. Neutropenia Grade 3 or 4 occurred in 5/52 (10%) of patients. The 1‐year overall survival was 83%, and the median overall survival was not reached after a median follow‐up time of 8.8 months. Factors including difference between involved and non‐involved free light chains (dFLC) ≥ 180 mg/L, N‐terminal pro‐brain natriuretic peptide (NTproBNP) ≥ 8500 pg/mL, glomerular filtration rate (GFR) < 20 mL/min/1.73 m^2^, and dialysis did not significantly impact overall survival. In total, nine patients deceased, six of whom deceased due to bacterial infections. Patients receiving immunoglobulin replacement therapy had a 2.01 lower risk of infections Grade 3 or 4 and a 6.14 lower risk of death. Our findings demonstrate the efficacy of teclistamab in a heavily diseased and pretreated cohort with AL amyloidosis and highlight the necessity of a concomitant immunoglobulin replacement therapy.

## Graphical abstract

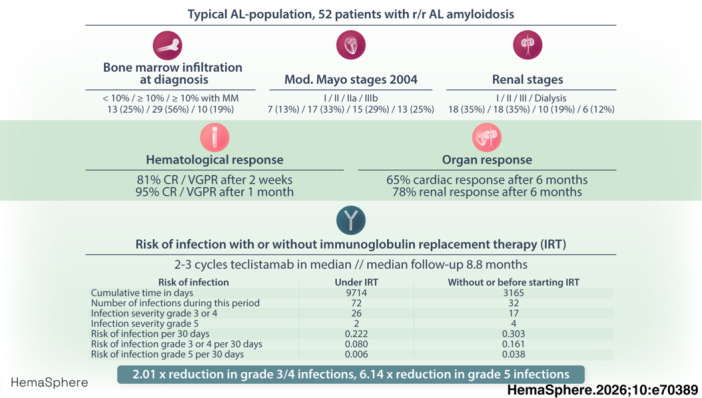

## INTRODUCTION

Systemic light chain (AL) amyloidosis is a rare acquired protein misfolding disorder with an incidence of about 5–13 cases per million person‐years.^1^ It is characterized by extracellular deposition of misfolded amyloidogenic immunoglobulin light chain fibrils secreted most often from clonal plasma cells in the bone marrow.^2^ Apart from the central nervous system, all organs can be affected, most commonly the heart and kidneys.^2^, ^3^ Clinical outcome of patients with AL amyloidosis is strongly associated with the severity of organ involvement, particularly cardiac involvement. Cardiac Mayo classifications from 2004 and 2012 were established to assess the severity of the disease.^4^, ^5^, ^6^

In 2021, daratumumab, an IgGκ monoclonal anti‐CD38 antibody, in combination with bortezomib, cyclophosphamide, and dexamethasone, was approved as first‐line therapy for patients with AL amyloidosis following the positive results from the randomized Phase III clinical trial (ANDROMEDA).^7^ In relapsed or refractory cases, there is no approved treatment for patients with AL amyloidosis. Consequently, treatment in relapse is based on more or less tolerable and dose‐reduced regimens derived from the treatment of multiple myeloma.^8^, ^9^, ^10^, ^11^, ^12^, ^13^

Teclistamab is a bispecific antibody that targets the CD3 receptor complex on T‐cells and the B‐cell maturation antigen (BCMA) receptor on plasma cells. T‐cell activation and the production of cytokines lead to lysis of BCMA‐positive cells. Teclistamab is approved for patients with multiple myeloma after three lines of treatment, based on the data from the MajesTEC‐1 study,^14^ which have been confirmed in a real‐world cohort.^15^ In AL amyloidosis, only a limited number of reports describing teclistamab efficacy have been published, mostly involving small patient cohorts and brief follow‐up periods. In addition, the populations published to date were mainly treated within the scope of the approval for multiple myeloma, which does not correspond to the actual AL patient population and therefore essentially represent a biased subpopulation.^16^, ^17^, ^18^, ^19^

The objective of this, to date, largest retrospective multicenter study, was to analyze the efficacy and prognosis of treatment with teclistamab in relapsed/refractory AL amyloidosis, particularly, the depth of hematologic responses achieved, as well as the corresponding organ responses. Furthermore, we aimed to evaluate side effects such as infections and the relevance of a concomitant immunoglobulin replacement therapy (IRT) due to the severe B‐cell suppression.

## METHODS

This retrospective study enrolled 52 patients with systemic AL amyloidosis, who received teclistamab after at least one prior treatment line from June 2022 until August 2025 at 11 centers and sites in Germany (Essen, Heidelberg, Oldenburg, Berlin, Hamburg, Cologne, Dresden, Frankfurt, Freiburg, Münster, and Würzburg). All patients have given their written consent to therapy. Data cut‐off was on September 30, 2025. Two patients from Essen and one from Heidelberg were previously reported in a case series on teclistamab with a shorter follow‐up period.^16^ In 44/52 cases (85%), teclistamab was administered outside the approved myeloma indication. The data were carefully extracted from clinical documentation as part of regular patient care. During the dose escalation phase, the ICE score^20^ was determined daily, and vital signs (blood pressure, pulse, oxygen saturation, and temperature) were documented at least every 8 h.

The diagnosis of AL amyloidosis was made according to standard criteria (tissue evidence of amyloid deposits from light chains and evidence of plasma cell dyscrasia), and the possible association with concomitant multiple myeloma was assessed according to the IMWG criteria^21^ with the exception that an free light chain (FLC) ratio > 100 was not considered a myeloma‐defining event. The Freelite® Test from Binding Site, Birmingham, United Kingdom, was used to measure free light chain levels. Organ involvement was assessed as recommended by the 10th International Symposium on Amyloid and Amyloidosis.^22^

Patients with hematologic progression or organ progression in persistent plasma cell disease were included, regardless of their cardiac or renal stages. In the first cycle, step‐up dosing (0.06, 0.3, and 1.5 mg/kg) was performed in analogy to the treatment scheme for multiple myeloma. The first administration of the full dose of teclistamab in cycle one corresponded to Day 8. Each treatment cycle corresponds to a treatment period of 4 weeks. Dose reductions, for example, bi‐weekly or four‐weekly administrations due to achievement of a deep hematologic response, hematotoxicity, or the risk of infection were decided individually by the treating physicians. All patients except three received antiviral and *Pneumocystis jirovecii* pneumonia prophylaxis. Most patients (43/52) received IRT due to secondary immunoglobulin deficiency, which was initiated at different time points, usually with 20 g every 4 weeks. IRT dosing was adjusted at the discretion of the treating physicians.

Hematologic responses at Day 15 (±7), Day 29 (±7), 2 months (±14), 3 months (±14), and at 6 months (±28 days) were assessed as recommended by the International Society of Amyloidosis.^23^, ^24^ Patients with a difference between involved and non‐involved free light chains (dFLC) in the range of 20–50 mg/L were evaluated regarding low‐dFLC criteria.^25^, ^26^ Achievement of low‐dFLC partial response (PR) was considered equivalent to hematologic very good partial response (hVGPR). Patients with dFLC lower than 20 mg/L were excluded from evaluation for hematologic response. Flow‐MRD (minimal residual disease)^27^ was performed on bone marrow aspirates with a sensitivity of 10^−5^. Graded cardiac and renal responses at 3 and 6 months were assessed in patients with evaluable data.^28^, ^29^

Adverse events (AE) were graded according to the common terminology criteria for adverse events (CTCAE). Cytokine release syndrome (CRS) and immune effector cell‐associated neurotoxicity syndrome (ICANS) were graded according to the ASTCT consensus scale.^20^ To investigate the risk of infections with or without IRT, we calculated the sum of Grade 3/4 or Grade 5 infections (death) and divided this by the sum of patient‐days during which patients were or were not under IRT.

Overall survival (OS) was calculated from the date of treatment initiation to death or last follow‐up, and event‐free survival (EFS) from the date of treatment initiation to death or hematologic disease progression using the Kaplan–Meier method. The median follow‐up time was calculated based on the median time to censoring (reversed Kaplan–Meier). A univariate Cox regression analysis predicting possible prognostic factors for OS and EFS was performed. Corresponding hazard ratios (HRs), 95% confidence intervals (95% CIs), and two‐sided P values were reported. The statistical software applied to calculate the statistical significance was GraphPad PRISM.

## RESULTS

### Patient characteristics

This study included 52 patients with relapsed/refractory AL amyloidosis who were treated with at least one dose of teclistamab between June 2022 and October 2025 at 11 German centers. Patient characteristics and disease parameters are shown in Table 1. The median age of the cohort was 66.5 years. 19% (10/52) of patients had concomitant multiple myeloma with at least one myeloma‐defining criterion according to IMWG, whereby, in deviation from this, an FLC ratio > 100 was not considered a myeloma‐defining criterion. In more detail, at diagnosis, eight had bone lesions in computed tomography (CT) scan, five had bone‐marrow infiltration above 60%, two had hypercalcemia, and two had anemia. Fluorescence in situ hybridization (FISH) analyses at diagnosis were available for 46 patients; among these, 48% (22/46) were positive for gain 1q21, 33% (15/46) for t(11;14), and 11% (5/46) had one of the myeloma high‐risk abnormalities del17p, t(4;14), or t(14;16). Cardiac involvement was observed in 83% (43/52) of patients. According to the Mayo staging system (pre‐teclistamab), 29% (15/52) of patients were in Mayo Stage IIIa and 25% (13/52) in Mayo Stage IIIb. Renal involvement was present in 77% (40/52) of patients with 19% (10/52) in renal Stage III and 12% (6/52) requiring dialysis. Patients received a median of two prior lines of therapy; the median time from diagnosis to treatment with teclistamab was 33.8 months (range 3.4−163.1). All patients had previously been treated with daratumumab, 98% (51/52) had been exposed to bortezomib, and 56% had received immunomodulatory drugs. Only one patient had previously received an anti‐BCMA therapy with belantamab‐mafodotin. Further details on prior treatments are provided in Table S1.

**Table 1:** Patient characteristics and disease parameters in 52 patients with relapsed/refractory systemic light chain (AL) amyloidosis treated with teclistamab between 2022 and 2025 across Germany.

Age, median (range)	66.5 (45–83)
Gender, male/female	34 (65%)/18 (35%)
ECOG	
0/1	2 (4%)/31 (60%)
2/3	13 (25%)/6 (12%)
Involved light chain, lambda/kappa	37 (71%)/15 (29%)
dFLC (mg/L), median (range)	115 (0.2–1246)
Bone‐marrow infiltration at diagnosis	
<10%/≥10%/≥10% with MM defining event^a^	13 (25%)/29 (56%)/10 (19%)
FISH (*n* = 46)	
t(11;14) ≥ 10%, positive/negative	15 (33%)/31 (67%)
Gain 1q21 ≥ 10%, positive/negative	22 (48%)/24 (52%)
High risk,^b^ positive/negative	5 (11%)/41 (89%)
Organ involvement	
Heart	43 (83%)
Kidney	40 (77%)
Peripheral/autonomic nervous system	17 (33%)
Gastrointestinal tract	13 (25%)
Liver	12 (23%)
Other	13 (25%)
NTproBNP (pg/mL), *n* = 51, median (range)	3432 (76–36,261)
hsTnT (ng/mL), *n* = 46, median (range)	0.05 (0.011–0.2)
Mayo stages 2004	
I	7 (13%)
II	17 (33%)
IIIa	15 (29%)
IIIb	13 (25%)
GFR (mL/min/1.73 m^2^), *n* = 51, median (range)	40 (4.6–99)
Proteinuria g/24 h, *n* = 43, median (range)	1.4 (0–16)
Renal stages	
I	18 (35%)
II	18 (35%)
III	10 (19%)
Dialysis	6 (12%)
Prior lines of treatment, median (range)	2 (1–8)
1 line	18 (35%)
2 lines	11 (21%)
3 lines	8 (15%)
≥4 lines	15 (29%)

Abbreviations: dFLC, the difference between involved and non‐involved free light chain; ECOG, Eastern Cooperative Oncology Group Performance Status; FISH, fluorescence in situ hybridization; FLC, free light chain; GFR, glomerular filtration rate; hsTnT, high‐sensitive troponin T; MM, multiple myeloma; NTproBNP, N‐terminal pro‐brain natriuretic peptide.

^a^ IMWG criteria^21^ with the exception that an FLC ratio > 100 was not considered a myeloma‐defining event.

^b^ High risk is defined by the presence of deletion 17p, or t(4;14), or t(14;16).

### Teclistamab therapy and acute toxicities

Patients were treated according to the assessment of local centers, and decisions regarding the duration and frequency of therapy were made by the treating physicians. At the time of data cut‐off, 13 patients were still on therapy. The median duration of treatment was 5.3 cycles (range 0.25−42.5), with a median of 11.5 full doses of teclistamab administered (range 0−54). A detailed description of the actually applied application frequency per cycle can be seen in Figure S1.

A total of 51/52 (98%) patients were hospitalized to receive the step‐up doses with a mean hospitalization duration of 8.8 days (range 0−16). CRS occurred in 37% (19/52) of patients and lasted less than 1 day in 89% (17/19) of cases, with 12 classified as Grade 1, 3 as Grade 2, and 2 as Grade 3. Tocilizumab was administered in 12% (6/52) of patients (Table 2). ICANS was not observed in any case. Transient neutropenia Grade 3 or 4 occurred in 5/52 (10%) of patients after a median of 78 (72–168) days and lasted for a median of 14 days (5−71). In five cases, patients received granulocyte‐colony stimulating factor. In three cases, neutropenia prompted physicians to halve and in one case to quarter the frequency of dosing. During neutropenic episodes, one case of neutropenic fever occurred; no patient deceased during neutropenia. Furthermore, mild, transient decreases (Grades 1 and 2) in platelet counts and hemoglobin levels occurred. Red blood cell transfusion was administered in one patient; no other patients required transfusions or thrombopoietin receptor agonists.

**Table 2:** Teclistamab side effects including cytokine release syndrome, immune effector cell‐associated neurotoxicity syndrome, and hematotoxicities.

Occurrence of first CRS (yes/no)	19 (37%)/33 (63%)
CRS grades	
1/2	14 (74%)/3 (16%)
3/4	2 (11%)/0 (0%)
Dose level followed by occurrence of CRS	
1	12 (63%)
2	4 (21%)
3	3 (16%)
CRS duration	
<1 day	17 (89%)
≥1 day	2 (11%)
Tocilizumab administration (yes/no)	6 (12%)/46 (88%)
Occurrence of ICANS (yes/no)	0/52 (100%)
Neutropenia (yes/no)	6 (12%)/46 (88%)
Grade of neutropenia	
1/2	1 (17%)/0
3/4	3 (50%)/2 (33%)
Anemia (yes/no)	9 (17%)/43 (83%)
Grade of anemia	
1/2	5 (56%)/4 (44%)
3/4	0/0
Thrombopenia (yes/no)	8 (15%)/44 (85%)
Grade of thrombopenia	
1/2	5 (71%)/2 (29%)
3/4	0/0

Abbreviations: CRS, cytokine release syndrome; ICANS, immune effector cell‐associated neurotoxicity syndrome.

### Efficacy

**Figure 1:**
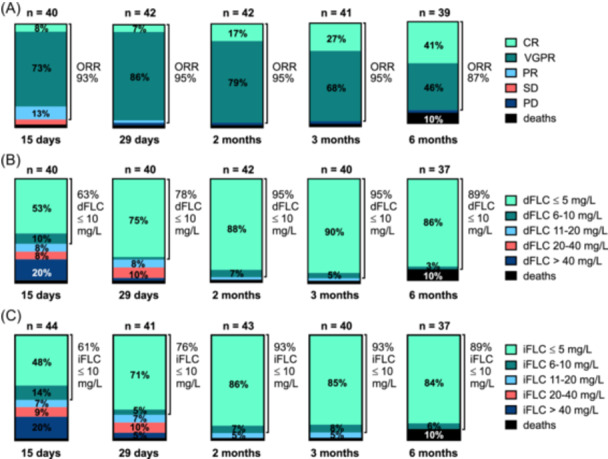
**(A) Hematologic response regarding International Society of Amyloidosis (ISA) response criteria, (B) difference between involved and non‐involved free light chains (dFLC), and (C) involved free light chain (iFLC) in relapsed/refractory systemic light chain (AL) amyloidosis treated with teclistamab.** Evaluable patients: 40, 42, 42, 41, and 39 at Days 15 and 29 as well as at 2, 3, and 6 months, respectively. Hematologic response was not evaluable due to low dFLC <20 mg/L in 6 patients; time point of analyses not reached in 2, 5, and 6 patients at 2, 3 and 6 months, respectively; missing data in 6, 4, 2, and 1 patients at Days 15, 29, and at 2 and 6 months, respectively. CR, complete response; hPR, hematologic partial response; ORR, overall response rate defined as hPR or better; PD, progressive disease; PR, partial response; SD, stable disease; VGPR, very good partial response.

Hematologic responses on Days 15 and 29, as well as at 2, 3, and 6 months following initiation of teclistamab therapy, are shown in Figure 1A. All but two patients responded to therapy; both non‐responders had multiple myeloma. One of them had del17p, was treated with teclistamab after three prior lines and deceased shortly after receiving the second step‐up dose due to hematologic and cardiac progression. Another patient showed no response after two full doses of teclistamab and was subsequently switched to talquetamab.

After 4 weeks of therapy (Day 29), 93% (39/42) of patients achieved complete hematologic response (hCR) or very good partial response (hVGPR). The proportion of patients achieving hCR increased from 7% (3/42) on Day 29 to 27% (11/41) at 3 months. Meanwhile, reduction of dFLC levels to ≤ 5 mg/L occurred in 75% (30/40) of patients on Day 29 and 90% (36/40) of patients on Day 85. Hematologic CR could not be determined despite negative immunofixation in some cases, as the uninvolved light chain was measured below the detection limit, and therefore, a ratio could not be calculated. As the involved light chain also declined to undetectable levels in these patients, a very deep response can be assumed; however, according to current response criteria, these responses cannot be classified as hCR. At 3 months, 19/28 of patients with hVGPR had an undetectable non‐involved light chain and therefore an incalculable light‐chain ratio. Of those, three patients were negative in serum and urine immunofixation and had undetectable involved light chains, three other patients with negative immunofixation had barely detectable involved light chain levels of 1.3, 1.4, and 2.8 mg/L. Therefore, presumably 6/28 of patients (21%) with hVGPR failed to meet hCR criteria solely due to the inability to calculate a light‐chain ratio. To further illustrate the depth of responses achieved, graphs for dFLC and involved free light chain (iFLC) are shown in Figure 1B,C.

Flow‐MRD with a sensitivity of 10^−5^ was performed in 24 patients at different timepoints, the earliest 7 days and the latest 385 days after the last dose of teclistamab. At the timepoints of MRD diagnosis, 13 patients were in hCR, 7 in hVGPR, and in 4 patients, hematologic response was not evaluable due to low dFLC < 20 mg/L. Only one patient had a positive MRD result. This patient had achieved hCR and was positive for t(14;16) and gain, 1q21.

In view of the rapid and profound hematologic response and therapy‐associated immunosuppression, the treating physicians decided to stop therapy in 34 patients. Of those, in five cases, termination of therapy was triggered by the occurrence of severe infections or poor general condition. In all 34 patients, in whom therapy was discontinued in hematologic remission, no cases of hematologic or organ progression were observed until data cut‐off after a median treatment‐free survival period of 5.7 months (range 0.5–20.8 months).

**Figure 2:**
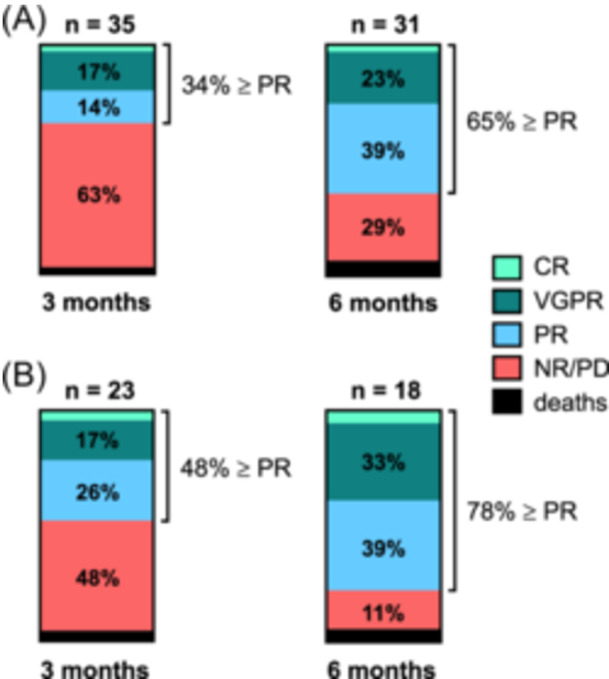
**(A) Graded cardiac response and (B) graded renal response in patients with relapsed/refractory systemic light chain (AL) amyloidosis treated with teclistamab.** (A) Evaluable patients: 35 and 31 at 3 and 6 months. Cardiac response was not evaluable because the time point of analyses was not reached in 5 and 7 patients at 3 and 6 months, missing data in 2 and 4 patients at 3 and 6 months, and low N‐terminal pro‐Brain natriuretic peptide (NTproBNP) < 650 pg/mL in one patient. (B) Evaluable patients: 23 and 18 at 3 and 6 months. Renal response was not evaluable because the time point of analyses was not reached in 4 and 7 at 3 and 6 months, missing data in 2 patients at 6 months, 5 patients on dialysis, and 8 patients without proteinuria before teclistamab. CR, complete response; NR, no response; PD, progressive disease; PR, partial response; VGPR, very good partial response.

Cardiac response rate (PR or better) in patients with cardiac involvement increased from 34% (12/35) at 3 months to 65% (20/31) at 6 months. In patients with renal involvement, renal response rate (PR or better) also increased from 48% (11/23) at 3 months to 78% (14/18) at 6 months (Figure 2A,B).

### Secondary immunoglobulin deficiency

All patients developed a secondary immunoglobulin deficiency due to B‐cell depletion during treatment. B‐cell counts were monitored regularly in 36 patients, generally on a monthly basis. A decline in B‐cell counts to values of 0–1/µL (reference 100−500/µL) was observed. During the observation period following discontinuation of teclistamab, seven patients showed signs of B‐cell regeneration. The median onset of regeneration, defined as the first B‐cell measurement above 5/µL, occurred 189 days (range 56–341 days) after the last teclistamab administration (Figure S2).

Substitution with intravenous or subcutaneous immunoglobulins (IRT) was performed in 83% (43/52) of patients; 17 patients received IRT preemptively, whereas 26 patients after infections had already occurred. In nine patients, in whom IRT was not given, four deceased of bacterial sepsis. IRT was initiated after a median of 42 days after starting teclistamab (range 0–322 days).

To assess the efficacy of IRT, we calculated the sum of Grade 3/4 or Grade 5 infections (death) and divided this by the sum of patient‐days during which patients were or were not under IRT. Patients without IRT were at an increased risk of Grade 3 or 4 infections (0.161 per 30 days) and an increased risk of septic death (0.038 per 30 days). In contrast, patients receiving IRT were at a lower risk of Grade 3 or 4 infections (0.080 per 30 days) and a lower risk of fatal infections (0.006 per 30 days). This corresponds to a 2.01‐fold reduction in the risk of infections Grade 3/4 and a 6.14‐fold reduction in Grade 5 infections (Table 3).

**Table 3:** Risk of infection per 30 days with and without immunoglobulin replacement therapy in systemic light chain (AL) amyloidosis under treatment with teclistamab.

Risk of infection	Under IRT	Without or before starting IRT
Cumulative time in days	9714	3165
Number of infections during this period	72	32
Infection severity Grade 3 or 4	26	17
Infection severity Grade 5	2	4
Risk of infection per 30 days	0.222	0.303
Risk of infection Grade 3 or 4 per 30 days	0.080	0.161
Risk of infection Grade 5 per 30 days	0.006	0.038

Abbreviation: IRT, immunoglobulin replacement therapy.

### Overall survival and event‐free survival

**Figure 3:**
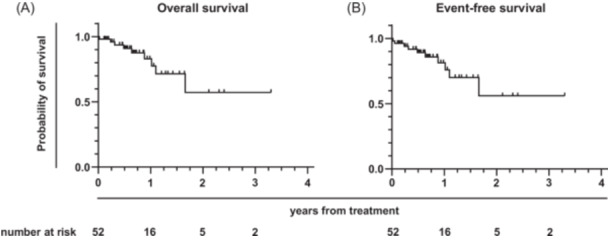
(A) Overall survival and (B) event‐free survival in 52 patients with relapsed/refractory systemic light chain (AL) amyloidosis treated with teclistamab.

Median OS and EFS were not reached after a median follow‐up time of 8.8 months (Figure 3A,B). The 12‐month OS and EFS rates were 83% and 81%, respectively. We performed a Cox regression analysis to investigate whether any baseline, clonal, or organ parameters might have affected OS or EFS. Factors including dFLC ≥ 180 mg/L, high‐risk cytogenetics, presence of multiple myeloma, NTproBNP ≥ 8500 pg/mL, glomerular filtration rate < 20 mL/min/1.73 m^2^, and dialysis dependence did not significantly impact OS or EFS (Table 4).

**Table 4:** Univariate Cox regression analyses for overall survival and event‐free survival in patients with relapsed/refractory systemic light chain (AL) amyloidosis treated with teclistamab.

Variable	*n*	OS			EFS		
		HR	95% CI	P	HR	95% CI	P
Age ≥ 65 years	52	0.64	0.14–3.25	0.56	0.80	0.20–3.92	0.77
Gender (reference: male)	52	2.20	0.52–9.41	0.27	1.74	0.43–6.62	0.42
AL amyloidosis with MM versus without MM^a^ (reference)	52	0.61	0.08–3.09	0.57	1.09	0.20–4.62	0.91
dFLC ≥ 180 mg/L.	52	0.82	0.16–3.54	0.79	1.17	0.27–4.63	0.79
1q21 ≥ 10%	46	1.50	0.28–6.93	0.63	1.46	0.28–6.93	0.63
t(11;14) ≥ 10%	46	0.84	0.12–3.93	0.83	0.84	0.12–3.93	0.83
High risk Del 17p, t(4;14), t(14;16)	46	4.68	0.65–24.12	0.11	4.68	0.65–24.12	0.11
Mayo IIIb versus other stages	52	3.57	0.84–15.13	0.08	2.82	0.70–10.69	0.15
NTproBNP ≥ 8500 pg/mL	51	3.86	0.91–16.35	0.07	3.05	0.75–11.57	0.11
hsTnT ≥ 0.05 ng/mL	46	3.19	0.49–62.15	0.24	1.67	0.35–11.89	0.54
Renal stages	52						
II versus I		3.86	0.57–75.82	0.18	4.91	0.79–94.37	0.25
III versus I		3.96	0.37–86.45	0.25	3.87	0.37–83.96	0.09
Dialysis	52	1.53	0.08–9.05	0.71	1.28	0.07–7.23	0.71
Proteinuria ≥ 5 g/24 h	45	1.74	0.23–10.53	0.55	1.31	0.18–6.72	0.76
GFR < 20 mL/min/1.83 m^2^	52	1.25	0.06–7.47	0.84	1.02	0.05–5.84	0.98

Abbreviations: CI, confidence interval; EFS, event‐free survival; FLC, free light chain; GFR, glomerular filtration rate; HR, hazard ratio; hsTnT, high‐sensitive troponin T; MM, multiple myeloma; NTproBNP, N‐terminal pro‐brain natriuretic peptide; OS, overall survival.

^a^ According IMWG criteria,^21^ with the exception that an FLC ratio > 100 was not considered a myeloma‐defining event.

## DISCUSSION

To date, this is the largest retrospective study that analyzed the efficacy and toxicity of teclistamab in relapsed/refractory systemic AL amyloidosis. In contrast to previous studies,^16^, ^17^, ^18^, ^19^ the majority of patients in our study were treated outside the approved myeloma indication. Our study reports, for the first time, the use of teclistamab in a classic AL population, with only 19% of patients having associated multiple myeloma. We demonstrate rapid achievement of hematologic responses, often to undetectable levels, which makes the evaluation of hematologic responses more challenging. Furthermore, this is the first study to highlight the critical role of IRT in reducing the risk of severe infections and septic death during teclistamab treatment in AL amyloidosis.

Patients achieved rapid and profound hematologic responses, with an overall hematologic response rate of 93% by Day 15 and 81% achieving hCR or hVGPR (Figure 1A). These findings are consistent with previously published case series reporting smaller patient numbers.^16^, ^17^, ^18^, ^19^ In patients who reached a hVGPR at 3 months, the inability to calculate the light‐chain ratio due to the undetectable uninvolved light chain was the reason why hCR could not be determined in 21% of cases. While standard International Society of Amyloidosis (ISA) criteria^23^, ^24^ are validated, well‐established, and widely used, our observations should be considered hypothesis‐generating and suggest that, in patients receiving teclistamab, their interpretation may warrant additional consideration, particularly when distinguishing hCR from hVGPR, as this treatment affects both involved and uninvolved light chains. In this context, more sensitive approaches, such as mass spectrometric quantification of light chains,^30^ could potentially provide complementary information due to their higher sensitivity; however, their role in this specific setting remains to be defined and requires validation.

As expected, the profound hematologic responses translated into increased organ responses; at 6 months, 65% of patients achieved a cardiac response and 78% a renal response (Figure 2A,B). Compared with previously published second‐line or later therapies—including ixazomib/dexamethasone,^9^ bendamustine,^10^, ^31^ venetoclax,^13^ lenalidomide,^12^, ^32^ and pomalidomide/dexamethasone,^11^ teclistamab demonstrated superior hematologic and organ response rates, comparable to those reported for BCMA CAR T‐cell therapies.^33^, ^34^

The 12‐month OS rate was 84% and the median OS has not been reached, despite our cohort representing one of the highest risk cohorts reported to date, characterized by a high proportion of advanced Mayo stages (29% IIIa and 25% IIIb), dialysis (12%, 6/52) proteinuria > 5 g (21%, 11/52), and a high proportion of +1q21 (48%, 22/46).^35^ These survival data are comparable to, or even better than, those reported in other relapse studies, in which treatments typical for multiple myeloma were used.^9^, ^10^, ^11^, ^12^ Only one study of treatment with venetoclax showed better results in patients with t(11;14).^13^ However, Mayo stages/renal stages were not reported in detail, and only 6% harbored +1q21, making it difficult to assess comparability, also via cross‐trial comparison. In our view, the excellent survival data in our study are the result of very rapid and deep hematologic responses combined with good acute tolerability of the therapy, as it is specifically targeted against plasma cells and does not directly affect other organs.

Overall, CRS occurred less frequently compared to the MajesTec‐1 study (37% vs. 73%), and all patients were able to be fully dosed. In contrast to the MajesTec‐1 study, ICANS did not occur at all in our cohort (0% vs. 15%). Hematotoxicity was also much less frequent: Grade 3/4 neutropenia (10% vs. 64%), anemia (0% vs. 37%), and thrombocytopenia (0% vs. 21%). The lower hematotoxicity in our AL amyloidosis cohort compared to the multiple myeloma trial may be explained by differences in the number of prior treatment lines, teclistamab dosing, plasma cell infiltration in bone marrow, which is typically higher in multiple myeloma, and the cumulative toxicity of previously administered therapeutic agents before teclistamab. Overall, teclistamab demonstrated very good tolerability in AL amyloidosis and could even be administered to severely ill patients, for example, those with Mayo Stage IIIb, nephrotic range proteinuria, or dialysis.

In our view, the main clinical problem is immunosuppression as a result of profound and prolonged B‐cell depletion, which is mostly related to the therapy with teclistamab. All patients showed a reduction of B‐cells to 0–1/µL, which led to secondary immunoglobulin deficiency. In seven patients, the beginning of B‐cell regeneration occurred at a median of 189 days (range 56–341 days) after the last dose of teclistamab, illustrating the long duration of immunosuppression. In total, 42% of patients suffered Grade 3 or 4 infections, comparable to the MajestTec‐1 study.^14^ However, in our cohort, a total of six patients deceased of bacterial sepsis; all patients were in deep hematologic remission, and a clear association with advanced Mayo or renal stages cannot be stated. Such cases were not explicitly listed in the MajesTec‐1 study, but it was reported that 12/165 (7%) patients deceased of COVID‐19. All patients in our cohort developed hypogammaglobulinemia; 43/52 of patients (83%) received IRT, compared to only 39% in the MajesTec‐1 study. After calculating the risk of infections Grade 3, 4, or 5 occurred per 30 days with/without IRT, we show that IRT reduced the risk of a Grade 3 or 4 infection by a factor of 2.01 and the risk of a Grade 5 infection by a factor of 6.14. Our data clearly indicate that IRT is essential and effective in patients receiving teclistamab. A further analysis, which also took into account the presence of nephrotic proteinuria and/or the need for dialysis, suggests that IRT also reduces the risk of infection in these conditions (Table S2). A previous analysis of multiple myeloma patients treated with anti‐BCMA bispecific antibody therapies also showed the importance of IRT.^36^

Two patients in the cohort deceased of bacterial sepsis despite IRT. In both cases, the patients presented with their infections outside the center, and antibiotic therapy was started too late in both cases, only when sepsis was already advanced. Even though the data available is sparse, additional antibiotic prophylaxis, such as with fluorochinolones, could be discussed as a means of reducing infections/mortality. However, this would in all likelihood lead to increased resistance development and the selection of multi‐resistant pathogens; in our view, consistent implementation of IRT and rapid initiation of appropriate antibiotic therapy in the case of infection is the correct strategy.

Given the high mortality associated with bacterial infections, future efforts should focus on developing strategies to reduce immunosuppression, either by limiting treatment duration or by reducing the dose over time. Based on our data, we believe that reducing treatment, particularly limiting its duration, is feasible. In 34 patients who were in hematologic remission and treatment with teclistamab was discontinued, no progression was observed after a median follow‐up of 5.7 months. These concepts are already being pursued in the ongoing EMN 40 study (ClinicalTrials.gov ID NCT06649695) in which treatment is limited to 6 months, and the teclistamab dose over time is approximately halved.

Our study has several limitations. Most notably, the retrospective design and the unavailability of certain clinical parameters or response data in some patients. In addition, treatment schedules were determined at the discretion of the treating physicians and did not follow a strict protocol. Another limitation is the relatively short follow‐up time of 8.8 months. Nevertheless, even at this early stage, our study is highly relevant, as it demonstrates rapid treatment responses in a relatively large cohort, and it is the first to address the main complication of teclistamab therapy, in particular, the occurrence of severe and even fatal (Grade 5) infections. We also suggest an effective solution through a concomitant IRT, which should be initiated early, to overcome and reduce the rate of severe infections. These findings are of outstanding importance for many treating physicians, who are already using teclistamab, both in multiple myeloma and AL amyloidosis.

Treatment with teclistamab is well tolerated and feasible in a broad range of patients with AL amyloidosis, including those with severe pre‐existing conditions (Table 4). Hematologic and organ response rates previously considered unattainable are being achieved, this is partially overshadowed by a high risk of infection‐related mortality. The administration of IRT appears to be mandatory to reduce the risk of mortality. Alternative treatment schedules and dosing strategies aimed at reducing immunosuppression should be evaluated in future studies.

## AUTHOR CONTRIBUTIONS

**Alexander Carpinteiro**: Conceptualization; methodology; investigation; validation; formal analysis; supervision; visualization; project administration; resources; writing—original draft; writing—review and editing; data curation; software. **Christoph Kimmich**: Conceptualization; methodology; data curation; investigation; validation; formal analysis; writing—original draft; writing—review and editing. **Despina Trajanova**: Conceptualization; investigation; writing—original draft; methodology; validation; writing—review and editing; formal analysis; data curation. **Ute Hegenbart**: Data curation; investigation; writing—review and editing. **Timon Hansen**: Investigation; writing—review and editing; data curation. **Udo Holtick**: Investigation; writing—review and editing; data curation. **Vera von Landenberg‐Roberg**: Writing—review and editing; data curation; investigation. **Stephan Rainer Bohl**: Investigation; writing—review and editing; data curation. **Raphael Teipel**: Investigation; writing—review and editing; data curation. **Ivana von Metzler**: Investigation; writing—review and editing; data curation. **Monika Engelhardt**: Investigation; writing—review and editing; data curation. **Evgenii Shumilov**: Writing—review and editing; investigation; data curation. **Maximilian Steinhardt**: Investigation; writing—review and editing; data curation. **Hans Christian Reinhardt**: Investigation; writing—review and editing; data curation. **Sara Oubari**: Conceptualization; methodology; software; data curation; investigation; validation; formal analysis; supervision; visualization; project administration; resources; writing—original draft; writing—review and editing. **Stefan Schönland**: Conceptualization; investigation; writing—original draft; methodology; validation; visualization; writing—review and editing; software; formal analysis; project administration; data curation; supervision; resources.

## CONFLICT OF INTEREST STATEMENT

A.C. received consulting and/or lecture fees from Alexion, Alnylam, Amgen, GSK, Johnson&Johnson, Pfizer, Sanofi, and Takeda, and travel and congress participation grants from Johnson&Johnson. C.K. received consulting, travel, and lecture fees from Amgen, AstraZeneca, Gilead, GSK, Johnson&Johnson, Oncopeptides, Pfizer, Sanofi‐Aventis, Sobi, and Takeda. D.T. has no conflicts of interest to declare. U.H. received honorarium for talks: Johnson&Johnson, Pfizer, Alnylam, and Akcea. Financial support for congress participation: Johnson&Johnson, Prothena, and Pfizer. Advisory boards: Pfizer, Prothena, and Johnson&Johnson. Financial sponsoring of Amyloidosis Registry: Prothena and Johnson&Johnson. T.H. received consulting and/or lecture fees from Alexion, Alnylam, Amgen, GSK, Johnson&Johnson, Menarini Stemline, Oncopeptides, Pfizer, and Sanofi. U.Ho. received honoraria from Amgen, BMS/Celgene, GSK, Johnson&Johnson, Jazz, Pfizer, Sanofi‐Aventis, and Takeda. V.v.L.‐R. received consulting, travel, and lecture fees from Johnson&Johnson, Takeda, and Sanofi. S.R.B. received consulting, travel, and/or lecture fees from Amgen, Johnson&Johnson, Sanofi, Pfizer, AbbVie, and GSK. R.T. received consulting and/or lecture fees from AbbVie, Amgen, BMS, Gilead, GSK, Johnson&Johnson, Oncopeptides, Pfizer, Sanofi, Stemline, and Takeda, and received research grants from Johnson&Johnson. I.v.M. received consulting, travel, and lecture fees from AbbVie, Johnson&Johnson, BMS, GSK, Sanofi, Oncopeptides, Stemline, Amgen, and Pfizer. M.E. received lecture and travel support from von Amgen, GSK, Johnsosn&Johnson, Pfizer, Sanofi, and Takeda, all unrelated to this study and data. E.S. has no conflicts of interest to declare. M.S. received research funds from Bayer, travel funds from Johnson&Johnson, and served in a consulting role for Johnson&Johnson and Alexion. H.C.R. received consulting and lecture fees from AbbVie, Roche, KinSea, Vitis, Cerus, Lilly, Novartis, Takeda, AstraZeneca, Vertex, and Merck and research funding from AstraZeneca and Gilead Pharmaceuticals. H.C.R. is a co‐founder of CDL Therapeutics GmbH. S.O. received travel and congress participation grants from Johnson&Johnson and Alexion. S.S. received research support from Johnson&Johnson, Prothena, and Sanofi; participation in advisory boards for Johnson&Johnson, Telix, Sobo, and Prothena; received honoraria from Johnson&Johnson, Takeda, Pfizer, and Prothena; and travel and congress participation grants from Johnson&Johnson, Prothena, Celgene, Binding Site, and Jazz. All are unrelated to this study and data.

## ETHICS STATEMENT

The study was approved by the Ethics Committee of the Medical Faculty of the University of Duisburg‐Essen (approval number 25‐12518‐BO) and conducted in accordance with the Helsinki Declaration of 1975, as revised in 2013.

## Supporting information

Supporting Information.

## Data Availability

The data that support the findings of this study are available on request from the corresponding author. The data are not publicly available due to privacy or ethical restrictions.
