# Supplementary material for: Teclistamab in relapsed/refractory light chain amyloidosis: A retrospective multicenter study by the German Society for Amyloid Diseases
Source: Hemasphere. 2026 Jun 16;10(6):e70389. doi: 10.1002/hem3.70389 (PMC13270341; doi:10.1002/hem3.70389)
Supplement: Supplementary file 1 — Supporting Information. [file HEM3-10-e70389-s001.pdf]

Supplementary

Tables

Table S1. Prior treatment lines of patients with relapsed/refractory systemic AL amyloidosis treated with teclistamab between 2022 and 2025 across Germany.

Prior treatment classes	Exposed/refractory patients (%)
**IMiDs**	
Lenalidomide	24 (46%)/17 (33%)
Pomalidomide	13 (25%)/12 (23%)
Thalidomide	1 (2%)/0 (0%)
**Proteasome inhibitors (PI)**	
Bortezomib	51 (98%)/25 (48%)
Cafilzomib	3 (6%)/3 (6%)
Ixazomib	1 (2%)/1 (2%)
**Anti-CD38-antibody**	
Daratumumab	52 (100%)/39 (75%)
**Anti-BCMA-antibody**	
Belantamab mafodotin	1 (2%)/1 (2%)
**BCL-2 inhibitors**	
Venetoclax	4 (8%)/2 (4%)
**High dose melphalan**	9 (17%)/2 (4%)
**Triple-class***	29 (56%)/13 (25%)
**Penta-class**†	3 (6%)/3 (6%)

BCL-2, B cell lymphoma 2 protein; BCMA, B cell maturation antigen; and IMiD, immunomodulatory drugs.

*Triple-class exposed: defined by exposition to IMiD, PI and CD38-antibody.
†Penta-class exposed: defined by exposition to two IMiDs, two PIs and CD38-antibody.

Table S2. Risk of infection regarding proteinuria.

	Proteinuria < 3g		Proteinuria ≥ 3g or dialysis	
Risk of infection	**without or before starting IRT**	**under IRT**	**without or before starting IRT**	**under IRT**
Cumulative time in days	1609	6498	1507	3075
Number of infections	16	45	15	26
Infection severity grade 3 or 4	9	18	8	10
Infection severity grade 5	2	1	2	1
Risk of infection per 30 days	0.29	0.21	0.298	0.253
Risk of infections grade 3/4 per 30 days	0.17	0.08	0.159	0.097
Risk of infection grade 5 per 30 days	0.04	0.005	0.039	0.009

IRT; Immunglobulin replacement therapy.

Figures

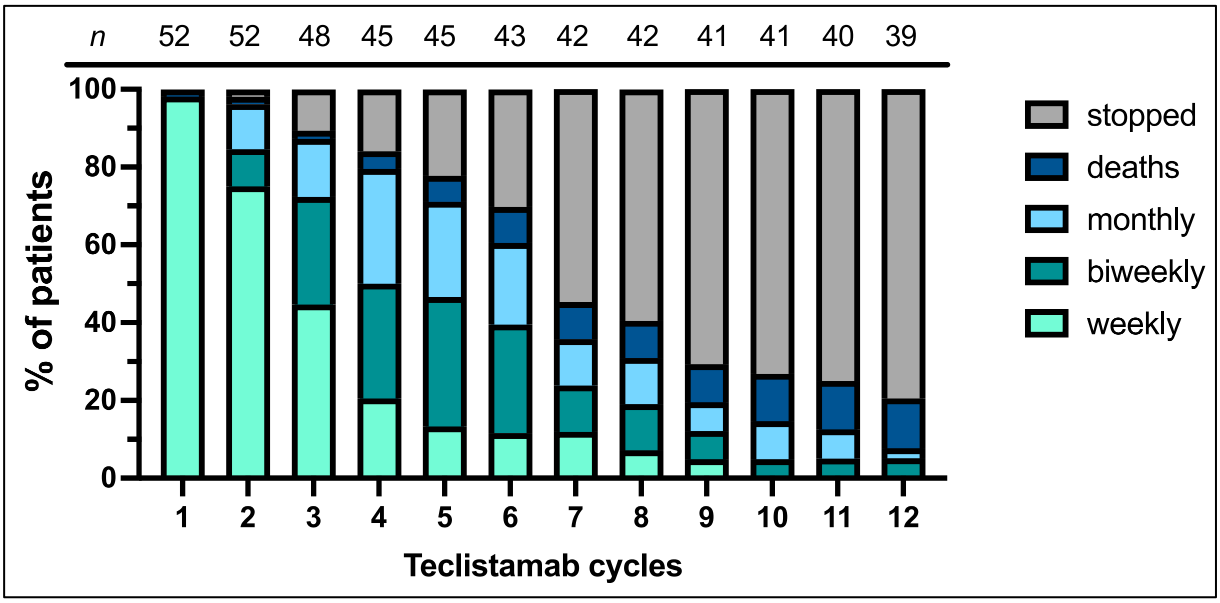

Figure S1. Detailed description of the applied frequency of teclistamab per cycle.

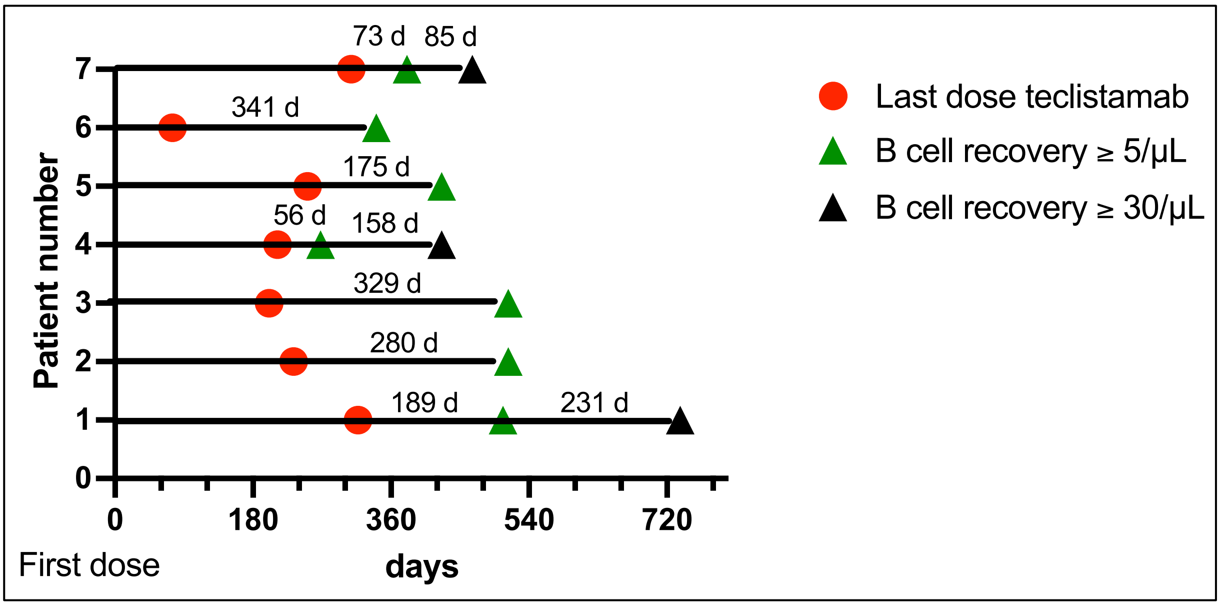

Figure S2. Timeplot for B-cell recovery ≥ 5/µL and ≥ 30/µL (reference 100-500/µl) in patients with relapsed/refractory systemic AL amyloidosis treated with teclistamab.
